# Supporting frail seniors through a family physician and Home Health integrated care model in Fraser Health

**DOI:** 10.5334/ijic.1069

**Published:** 2014-03-10

**Authors:** Grace Park, Diane Miller, George Tien, Irene Sheppard, Michael Bernard

**Affiliations:** Home Health, Fraser Health, Surrey, BC, Canada; Primary Health Care & Aboriginal Health, Fraser Health, Surrey, BC, Canada; Lead, Evaluation, Integrated Primary Community Care, Fraser Health, Surrey, BC, Canada; Home Health Program, Fraser Health, Surrey, BC, Canada; North Vancouver, BC, Canada

**Keywords:** integrated care for frail seniors, family physician–case manager partnership, home health–primary care integration, integrated primary and community care

## Abstract

**Background:**

A major effort is underway to integrate primary and community care in Canada's western province of British Columbia and in Fraser Health, its largest health authority. Integrated care is a critical component of Fraser Health's planning, to meet the challenges of caring for a growing, elderly population that is presenting more complex and chronic medical conditions.

**Description of integrated practice:**

An integrated care model partners family physicians with community-based home health case managers to support frail elderly patients who live at home. It is resulting in faster response times to patient needs, more informed assessments of a patient's state of health and pro-active identification of emerging patient issues.

**Early results:**

The model is intended to improve the quality of patient care and maintain the patients’ health status, to help them live at home confidently and safely, as long as possible. Preliminary pilot data measuring changes in home care services is showing positive trends when it comes to extending the length of a person's survival/tenure in the community (living in their home vs. admitted to residential care or deceased).

**Conclusion:**

Fraser Health's case manager–general practitioner partnership model is showing promising results including higher quality, appropriate, coordinated and efficient care; improved patient, caregiver and physician interactions with the system; improved health and prevention of acute care visits by senior adult patients.

## Introduction

Fraser Health is one of Canada's largest and fastest growing healthcare authorities, providing public health care services for 1.6 million people residing in a southwestern British Columbia, Canada. One of five provincial health authorities, Fraser Health administers a $2.6 billion budget, employs 26,000 people, oversees 12 acute care hospitals and 7760 residential care beds, and delivers Home Health services to more than 14,000 clients. More than 1400 independent family physicians and specialists practise in the region.

Fraser Health's struggle to keep pace with a growing elderly population is an all-too familiar scenario played out in healthcare systems in other countries. An ageing population growing disproportionately faster than other demographic groups while presenting more complex and chronic medical conditions is challenging the healthcare system's ability to cope. Within Fraser Health's boundaries, the situation is exacerbated by daunting population growth figures. It helped little when pressures in Fraser Health's were highlighted in the national media stories about a popular coffee chain's in-hospital leased premises being commandeered to provide overflow space for a local hospital's crowded emergency room.

While this is an extreme example of a temporary fix, it did shine a light on an ongoing problem that many healthcare agencies face in effectively addressing profound changes in patient populations. Within the Fraser Health region, about 34 per cent of the population has multiple chronic conditions and use more than 80 per cent of the system's healthcare resources, with an increasing number of these individuals requiring acute care services. The predicament is compounded by a shortage of family doctors.

The solution calls for a transformation in the way healthcare services are delivered, a challenge on which Fraser Health has been working for several years.

## Seeds of an integrated model

Seeds were sown four years ago following the success of an initiative pioneered by the Johns Hopkins School of Public Health [1,2]. There the growing problem of caring for older adults with chronic conditions was addressed by having a nurse based in a primary care office work with a number of physicians and other members of a care team to provide coordinated, patient-centred and cost-effective health care to 50–60 of the physicians’ chronically ill patients.

The nurse conducted in-home assessments, facilitated care planning, promoted patient self-management, monitored conditions monthly and coordinated the efforts of all health care professionals. The nurse also smoothed transitions among sites of care, educated and supported family caregivers, and facilitated access to community resources.

After 20 months, patients experienced on average, 30 per cent fewer home health care episodes and 21 per cent fewer hospital readmissions. The project produced even larger reductions in a subset of patients who received their primary care from one well-managed health system. Subsequent trials involving about 900 patients indicated improvements in the quality of patients’ care, improvements in family caregivers’ perception of quality and increased physician satisfaction with chronic care, and enhanced nurses’ job satisfaction.

### Fraser Health pilot

In 2007–08, emulating many features of the Johns Hopkins guided care model, Fraser Health formed the Tri-Cities Physicians Partnership, in a bid to improve the health care of a comparable senior adult population in a suburban area east of Vancouver. The outcomes were even more dramatic than those emerging from the Johns Hopkins pilot. There was a reported 33 per cent decrease in patient visits to the Emergency room and a 61 per cent decrease in the hospitalisation of a group of Home Health clients as well as increased client satisfaction. Buoyed by these results, Fraser Health set to work to design an approach that could be adopted throughout the system.

But rarely is true transformational change triggered by a single experiment or event. There were several major structural changes within the primary healthcare system concurrent with the Tri-Cities pilot, which laid the foundation for a new system of integrated primary and community care.

### Primary care renewal

Several international studies had been supporting the view that better primary care leads to better population health. One of the most influential studies was conducted in British Columbia.

In a landmark study [3], Dr. Marcus Hollander of Victoria examined usage data from more than 98,000 high-needs patients in BC and focused on diabetes and congestive heart failure. The results showed that people with chronic diseases who are regularly served by and attached to the same full-service family practice cost the healthcare system significantly less money those patients not closely attached to a family doctor. The study, published in Healthcare Quarterly in 2009, found that the average annual hospital costs for high-needs diabetes patients who were not attached to family practices were almost $17,000 annually. That compares, on average, to just $5900 for similar patients attached to a family practice. The highest needs patients with CHF cost the overall health system almost $30,000 if they were not attached to a family doctor, but just $12,000 if they were. The difference in costs were attributed in large part to the fact that patients without family doctors spend more days in hospital which greatly adds to the cost of the health care they receive. Hollander's study found that attachment to a family practice was the best predictor of the patient's overall health care costs and was more related to costs than other variables such as age.

The research showed that a reduced focus on episodic acute care as the main response to chronic and complex health issues, and a renewed emphasis on longitudinal primary care in an individual's medical journey would be an important shift to make to produce better results for patients, and to sustain a health care system under intense pressure.

One of the first footings to lay the foundation for primary care renewal in the Province was the Ministry of Health's introduction of the Primary Care Charter in 2005. It included the introduction of the General Practice Service Committee comprising representatives from the Ministry of Health and the BC Medical Association and physicians from the local area. The committee began by listening to physician concerns and working on developing operational solutions to their issues.

One of those issues was finding ways of developing business models that encouraged a deeper attachment of doctors to patients. This included compensating doctors for additional time spent on providing more comprehensive health care treatment for populations of patients with two or more chronic and complex medical conditions.

Also serving to foster and reinforce attachment of family physicians to patients was the formation of Divisions of Family Practice representing each of Fraser Health's 20 communities; networks in which family physicians can connect to discuss local primary care issues and provide input into healthcare decisions where there had none before. The process of establishing Divisions started in 2009 with Ministry funding to support administrative assistance and major initiatives undertaken by a Division to increase the attachment of patients to family physicians.

Accelerating the participation of family physicians was the creation of the Collaborative Services Committee attached to each Division of Family Practice; a formal structure that facilitates collaboration and decision-making between representatives from the Division family physicians, the Ministry and Fraser Health.

For its part, the provincial government committed to realigning its own structure to support an integrated primary and community care system, and establishing the policy environment in which health authorities could carry out this work. It also adopted the American-based Institute for Healthcare Improvement critical objectives or “Triple Aim” as performance measures for the BC healthcare system. Those are to:

- Improve the health of the population;
- Enhance the patient and provider experience of care (including quality, access, and reliability);
- Reduce, or at least control, the per capita cost of care.

### Organisational changes

Fraser Health undertook its own organisational changes to fuel the strengthening of primary care and integration of community services. To break down historical silos, Fraser Health looked to create new collaborative structures. One such move involved placing acute care medicine, community care and primary care under a single executive leader, encouraging synergies between previously disparate areas, but which often served common populations. These changes are credited with helping Fraser Health to be the first in the province to pioneer the “Home Is Best” strategy, in which acute care Medicine and the Home Health community programme work together to facilitate seniors recovering their health in their own homes after a hospital stay.

What also helped was a consolidation of some home support services, which achieved administrative savings through economies of scale and other efficiencies. These savings freed up funding to be reallocated to Home Health in some redesign work necessary for integration with primary care.

### Integration of primary and community care: case manager–general practitioner partnership

With these supportive features in place, Fraser Health developed a strategy in 2010 for its Home Health, Mental Health and Substance Use and Older Adult programmes to work with family physicians in Divisions of Family Practice, and to test an integrated primary and community care model that could support a frail elderly priority population.

The first stream of integration was implementation of the Home Health case manager – general practitioner partnership initiative (also called ‘case manager–GP partnership’).

Traditionally, Home Health case managers had been assigned to long-term care clients on a geographical basis receiving referrals from family physicians, family members and a variety of social agencies. They would undertake assessments of the clients’ needs and develop care plans. Communication between case managers and family physicians was sporadic and challenging at the best of times. Case managers frequently found it difficult if not impossible to reach a physician by phone to discuss a client's situation. Faxed messages became the norm. For their part, physicians were frustrated by having to deal with a faceless service. With little feedback, physicians were left to wonder if what they had requested of the system for their patients was getting done.

With the introduction of the case manager–general practitioner partnership initiative in 2010, case managers were moved from geographic assignments and partnered with family doctors to collaboratively support common patients; specifically frail elderly patients with medical issues, living in their own homes and requiring long term support.

As part of the new process, the Home Health case managers and family physician partners agreed to regularly hold ‘case conferences’ to discuss the patient's needs and progress, share information and coordinate care.

Doctors were soon pleasantly surprised to find that case managers gather intelligence that they do not have. Much of this additional information results from case managers employing the resident assessment instrument, a thorough process that assesses not only the client's medical status but also their functional status, mobility, home environment and limitations, and social status among other conditions. Given the nature of the target population, the value of the information doctors received was greatly enhanced. For instance, knowing that adult children may be burning out from caring for an increasingly frail parent can be a critical social issue in considering their future care.

Physicians and case managers have seen other direct benefits of face-to-face consultation. Real time discussions can also lead to real time solutions when there is a timely assignment of resources to a particular patient's situation. As well, case conferences give both Home Health case managers and family physicians an opportunity to assess a patient's status from a number of different perspectives and to anticipate events that might lead to an emergency room visit or a hospital stay, key goals of integration in this initiative.

The process of gaining the support of both case managers and doctors has not been without its challenges. Initially some case managers resisted the changes. They foresaw an erosion of the autonomy they had enjoyed previously and believed that their partnership with doctors may be a hierarchical one reminiscent of former days as nurses in a hospital setting where doctors often held the balance of power. Some case managers, while welcoming a return to their clinical roots, feared that their new duties would be added to already burdensome administrative work. Steps were taken to address these concerns. Case managers were provided with a tool for identifying clients with lower needs or who were stable on their care plan. Those clients were then assigned to a “surveillance nurse” whose job was to follow up with clients by telephone to monitor their status on an ongoing basis and initiate actions as required to support the client to maintain their stability. Business support was also hired to handle time-consuming financial assessments and other paperwork which was neither clerical nor clinical but always time-consuming.

Another helpful benefit of the model has also been a testament to the power of collaboration on several levels.

### Provider observations

Before if I had a problem where my patient required care at home I would fax a message to Intake and sort of hope that someone at Home Health would pick it up and run with it. But I didn't know whom I would be communicating with and there wasn't really any one to call personally. You would just put the information into the system and hope it would eventually be taken care of.

(A White Rock family physician reflecting on his previous relationship with Home Health)

Interviews with Home Health case managers and general physicians reveal that the new approach is yielding benefits for providers as well as patients.

Dr. Bryan Prentice, who has a thriving family practice in South Surrey and White Rock, says that he finds this new relationship with Home Health works far better:

With this system, I sit down and talk with my case manager every four to six weeks. She (the case manager) often becomes aware of situations developing (with my patients) and brings me up to date with them.

While Dr. Prentice feels confident that he knows his patients well, he can think of at least one circumstance where the case manager brought him new information about how an elderly patient was doing. “She told me about issues he was having at home with his wife and family and an evolving dementia”. While the patient's chart shows he had not been compliant with his medication, the additional intelligence provided by the case manager enabled him to take more specific action and … “as a result, I called him in to deal with those things”.

Dr. Gordon Enns, a family physician in Chilliwack since 1991, has also seen definite advantages to the new system.

“The new system is superior for a number of reasons but there is one that always comes to mind for me – personal accountability”, he said. “I have her cell phone number on my phone now. And my staff has it on their speed dial at the office. So if we get a call that a patient needs a physiotherapist, you leave a cell phone message and the next day I will get a message back that Home Health has arranged for a physiotherapist to visit my patient at home or that an occupational therapist is doing an in-home assessment. At least I know it is dealt with rather than sending a fax into the black hole”.

Enns noted that the enhanced communication between physician and Home Health is a two-way street.

“Patients always go to their primary practitioner first, who they see as the access point for these things. But a referral to Home Health can come from a neighbour, a relative or a nurse”, which he notes opens the door to receiving vital information from other sources.

Kathy Churchill, a nurse for 30 years, much of it in intensive care, says she has worked the last seven years as a Home Health liaison in the hospital and the community, says the new Home Health system is much better than the old one:

I found the old system challenging. It was often difficult to get a hold of physicians if you needed certain things like medications or had concerns about the client. You would be dealing with many different doctors and they often didn't know who you are or what your role is.

She said she knows that both physicians and case managers felt frustrated in not knowing whether a request on behalf of a client was being implemented.

“There really is little understanding of the programmes and policies of Home Health, what criteria apply to seniors receiving care and what the role of Home Health really is.”

One issue that continually arises, she said, is that many physicians still do not know that Home Health does not provide housekeeping services (and has not for many years). There is a similar misunderstanding about Fraser Health-funded assisted living services. Many physicians do not know that seniors must be able to direct their own care (i.e. be cognitively intact) to qualify, or that more compromised seniors can get more care hours in their own home, if they need it, than they might through assisted living.

There are also some challenges under the new system, she said:

There is a heavier work load and more interaction with the client and their families is required. It used to be that we would go in and tell people what they need. Now there are expectations of greater client involvement and more frequent client contact. Now we are a partner in care with the family.

Cheryl Lindsay, a case manager in the City of Abbotsford, has worked for Home Health for five years, two of them in home support. She believes the strength of the new system is that physicians have just one person to deal with rather than a multitude of service providers.

Many family physicians who had only a passing knowledge of what Home Health does now have a better understanding of the breadth of services the case manager can call upon to help the client, including services for mental health issues, pharmacy, occupational therapy among others.

She also notes that some patients can “present very well (in front of a physician) but not necessarily be doing very well”, a fact that can be substantiated when Home Health visits a client's home:

For instance, is the senior really capable of driving? A case manager can help determine that by going into the home and doing a mini-assessment.

Lindsay said case managers working directly with doctors can also mitigate the risk of a visit to Emergency room by adjusting medication or attending to a wound at home. And when the patient must travel to hospital, Home Health case managers can also give Emergency Departments at local hospital a heads up by pointing out particular issues to attending physicians in advance of the client arriving there.

One of the best features is that medical needs can be better addressed at home, including some diagnostic testing, where the client is often more comfortable and relaxed. She also knows of cases where doctors have managed to expedite tests such as computerised tomography scans by working with a patient at home rather than having the patient go to an emergency room.

Perhaps the most persuasive case to be made for the new system is what follows from increased communication between physician and case manager, said Lindsay.

“When you are following up (about a client) and the physician sees this is working, it starts to build confidence and trust in Home Health and they are willing to share more information about their patient.” And what the client sees is that he or she has a team of people working for them:

The relationships we have with our physicians are important. The more we are open in our conversations and able to show that we really care about the work that physicians are doing, the more healthcare improves all around.

Gloria Anonsen, 85, of Abbotsford, says she found the new Home Health approach a major benefit for her husband Eric, for whom she has been the primary caregiver since he suffered a stroke 10 years ago. In fact, she found that with the integrated Home Health physician approach, it made a lot of sense for her and Eric to share the same doctor, thus building a greater attachment to their family physician:

It's great that she knows us, comes and sees us and talks to both of us. She has nothing to gain and we have a lot to gain. I know I can pick up the phone and say I need help, and I know she would do the best she could to help me.

## Early results

While case manager–general practitioner partnership model in Fraser Health started as a pilot in the eastern community of Chilliwack in 2010, it has since shown enough promise to be refined and expanded to five additional communities in the region.

While it is still early to measure statistically whether a partnership between Home Health and family physicians has reduced emergency room visits at Fraser Health's hospitals, evidence of its benefits is emerging.

In the community of Abbotsford, where the case manager–general practitioner partnership was introduced in May 2011, preliminary data measuring changes in home care services are showing positive trends when it comes to extending the length of a person's survival/tenure in the community (living in their home vs. admitted to residential care or deceased) [4].

The pilot data indicate that the cohorts “survive” (or fail) at different rates. At the 50th percentile (at the point when 50 per cent of the cohorts fail), the different cohorts have survived approximately the following number of days in the community:

- 2009 cohort – 223 days.
- 2010 cohort – 264 days.
- 2011 cohort – 463 days.

The case manager–general practitioner partnership may be partly the reason for the longer community tenure. The enhanced partnership is intended to improve the quality of patient care and maintain the patients’ health status, with a goal to help them live at home confidently and safely, as long as possible. One reflection of this is in the reduced use of acute care services or residential care by an individual, with the flipside being a longer community tenure which honours the wishes of most seniors to remain in their own home as they age. The data are consistent with the interpretation that the introduction of the case manager–general practitioner partnership in 2011 has resulted in keeping patients in the community for longer periods.

## Conclusion

The integrated care model that brings together family physicians with home health professionals is providing many benefits for elderly patients in Fraser Health communities. These include faster response times to patient needs, more informed assessments of a patient's state of health and pro-active identification of emerging patient issues.

At the heart of the partnership is a growing trust between Home Health staff and family physicians who are working together towards the same goals for a patient. Ultimately, family physicians have come to respect the case managers’ expertise and contributions; while most case managers had come to recognise that the physician is the constant in their patient's healthcare journey; and that a face-to-face, collaborative relationship where there was none before – the essence of integrated care – is resulting in better care for their patient.

## Appendix A

George Tien, Lead, Evaluation, Integrated Primary Community Care, Fraser HealthIrene Sheppard, Director Home Health programme, Fraser Health, Suite 400, Central City Tower, 13450-102nd Ave Surrey, BC V3T 0H1, Canada

**Background**

Home care services were changed in the target community beginning in 2011. The objective was to extend community tenure and delay admission to residential care.

**The pilot data**

The chart below shows that at the time the pilot data was collected, the 2011 cohort of clients appears to have already survived longer in the community compared to the 2009 and 2010 cohorts.

That is, the pilot data indicate that the cohorts “survive” (or fail) at different rates. At the 50th percentile (at the point when 50% of the cohorts fail), the different cohorts have survived approximately the following number of days in the community:

- 2009 cohort – 223 days
- 2010 cohort – 264 days
- 2011 cohort – 463 days

In the chart above,

- A steeper curve indicates shorter survival times, and a shallower curve, therefore indicates longer survival times.
- The darker areas of the curve indicate “censoring”. Censoring in this context generally means that a case is still in the community at the cut-off date for data collection. This means that their survival times are likely to be longer than the pilot data indicate. Note that in terms of the 2011 cohort, this should translate into an even shallower curve for the 2011 cohort if more time is allowed before initiating data collection.
- Note also that the rate of failure between the 2009 and the 2010 cohorts does not differ.
- However, the rates of failure between the 2011 and the 2010, as well as, between the 2011 and the 2009 cohorts do differ. These differences are reliable.

**Interpretation**

Given this pilot data, if the context remains unchanged, it is likely, that in this target community, this pattern will hold up over time and that as a whole, the length of community tenure of the 2011 cohort is expected to be longer compared to the 2009 and 2010 cohorts.
